# Genome-wide characterization of two-component system elements in barley enables the identification of grain-specific phosphorelay genes

**DOI:** 10.1186/s12870-025-06161-1

**Published:** 2025-02-17

**Authors:** Christian W. Hertig, Pravinya Devunuri, Twan Rutten, Götz Hensel, Jos H. M. Schippers, Bruno Müller, Johannes Thiel

**Affiliations:** 1 Department of Molecular Genetics, Leibniz Institute for Plant Genetics and Crop Plant Research (IPK), Seeland/OT Gatersleben, D-06466 Germany; 2 Department of Physiology and Cell Biology, Leibniz Institute for Plant Genetics and Crop Plant Research (IPK), Seeland/OT Gatersleben, D-06466 Germany; 3 Present address: University of Hohenheim, Schloss Hohenheim 1, Stuttgart, 70599 Germany; 4 Present address: Center for Plant Genome Engineering, Heinrich-Heine University, Düsseldorf, Germany; 5 Present address:, Microsynth AG, Balgach, 9436 Switzerland

**Keywords:** Two-component system, Barley, Genome, Expression analysis, Grain development, Cell types

## Abstract

**Background:**

The two-component system (TCS) serves as a common intracellular signal transduction pathway implicated in various processes of plant development and response to abiotic stress. With regard to the important cereal crop barley, only partial information about the occurrence of TCS signaling elements in the genome and putative functions is available.

**Results:**

In this study, we identified a total of 67 non-redundant TCS genes from all subgroups of the phosphorelay in the latest barley reference genome. Functional annotation and phylogenetic characterization was combined with a comprehensive gene expression analysis of the signaling components. Expression profiles hint at potential functions in vegetative and reproductive organs and tissue types as well as diverse stress responses. Apparently, a distinct subset of TCS genes revealed a stringent grain-specificity not being expressed elsewhere in the plant. By using laser capture microdissection (LCM)-based transcript analysis of barley grain tissues, we refined expression profiles of selected TCS genes and attributed them to individual cell types within the grain. Distinct TCS elements are exclusively expressed in the different maternal and filial cell types, particularly in the endosperm transfer cell (ETC) region. These genes are deemed to be selected in the domestication process of modern cultivars. Moreover, barley plants transformed with a synthetic sensor (*TCSn::GFP*) showed a high and specific activity in the ETC region of grains monitoring transcriptional output of the signaling system.

**Conclusions:**

The results provide comprehensive insights into the TCS gene family in the temperate cereal crop barley and indicate implications in various agronomic traits. The dataset is valuable for future research in different aspects of plant development and will be indispensable not only for barley, but also for other crops of the Poaceae.

**Supplementary Information:**

The online version contains supplementary material available at 10.1186/s12870-025-06161-1.

## Introduction

The two-component system (TCS) represents an ancient intracellular signal transduction pathway that functions alternatively to the mitogen-activated protein kinase (MAPK) cascade systems. TCS pathways involve phosphorylation of His and Asp residues in a modular arrangement to transmit signals from the membrane to the nucleus and subsequently, modulate cellular responses. TCS-mediated signal transduction pathways were firstly discovered in bacteria and initially described in *Escherichia coli* [1, 2]. The bacterial system comprises two signaling elements, a membrane-associated histidine kinase (HK) and a cytoplasmic response regulator (RR) that acts as transcriptional activator of downstream target genes. In eukaryotes, such as yeast, fungi and plants, an intermediate component -the histidine-containing transfer protein (HP)- which transfers the phosphate group from the receptor to the regulatory element, was integrated in the system, so is also assigned as multi-step phosphorelay (MSP) [3–5]. The membrane-bound hybrid HK gets autophosphorylated when a signal is perceived and the phosphate is transmitted to the receiver (REC) domain at the C-terminus of the protein, subsequently, the phosphate residue is transferred to HP, which moves to the nucleus and phosphorylates RR proteins that activate downstream targets in the nucleus.

In plants, TCS phosphorelays are involved in responses to abiotic stress, including shading [6], drought [7–9], salt [7] or cold [10] and the perception of plant hormones [11]. A prominent example is the mediation of cytokinin signals in *Arabidopsis*, but also direct or secondary interactions with other hormones, like ethylene, abscisic acid or auxins are described in the literature [12–15]. TCS phosphorelays are involved in various processes of plant development, particular cell and tissue differentiation, circadian mechanisms and induction of senescence [16–18]. A pivotal role in the regulation of meristematic activity in shoots and roots or vascular development has been demonstrated in several cases [19–21].

Genome-wide investigations of plant TCS elements were firstly described in the model plants *Arabidopsis* [22, 23] and rice [24] and a number of crop species like *Lotus* [25], soybean [26], maize [27], Chinese cabbage (*Brassica rapa*) [28], cucumber [29], tomato [30], melon [31] and banana [32]. With the advances in sequencing methods, overviews about TCS genes are now available for wild rice *Zizania latifolia* [33], *Sorghum* [7], *Brassica oleracea* [6] and sweet potato [34]. For the economically important temperate cereal crops wheat and barley, only partial overviews of TCS genes in the genome are currently available [9, 14].

Barley (*Hordeum vulgare* L.) is one of the oldest domesticated plants in the Fertile Crescent and can be regarded as a diploid model for polyploid wheat species. In barley, laser capture microdissection (LCM)-based RNA-sequencing revealed that the TCS is predominantly associated with initial endosperm differentiation and grain formation [35, 36]. Functional studies in barley grains have already elucidated the significant role of the *HISTIDINE KINASE 1* (*HvHK1*) for cell specification in the young endosperm [37]. Grain and endosperm development of cereals follows a coordinated and distinct pattern of cell- and tissue-specific differentiation that gives rise to the different cell types within the grain, developmental timing of grain formation and finally, size and yield of starchy edible grains. Understanding the molecular mechanisms triggering differentiation processes in grains and identification of key regulators is a prerequisite for improvement of yield and quality.

The objective of this study is to fill the gap of knowledge by presenting a comprehensive overview about TCS genes in barley as a representative of the Triticeae using the latest reference genome (Morex.V3) [38] and to collect data associated with putative functions in plant development. The article highlights information about gene sequences, protein/domain structures, genomic localization, phylogenetic relationships and gene expression in vegetative and reproductive tissues next to hormonal and/or stress responses. A particular focus is set on barley grain development.

## Results

### Genome-wide identification of barley TCS elements and localization on chromosomes

**Fig. 1:**
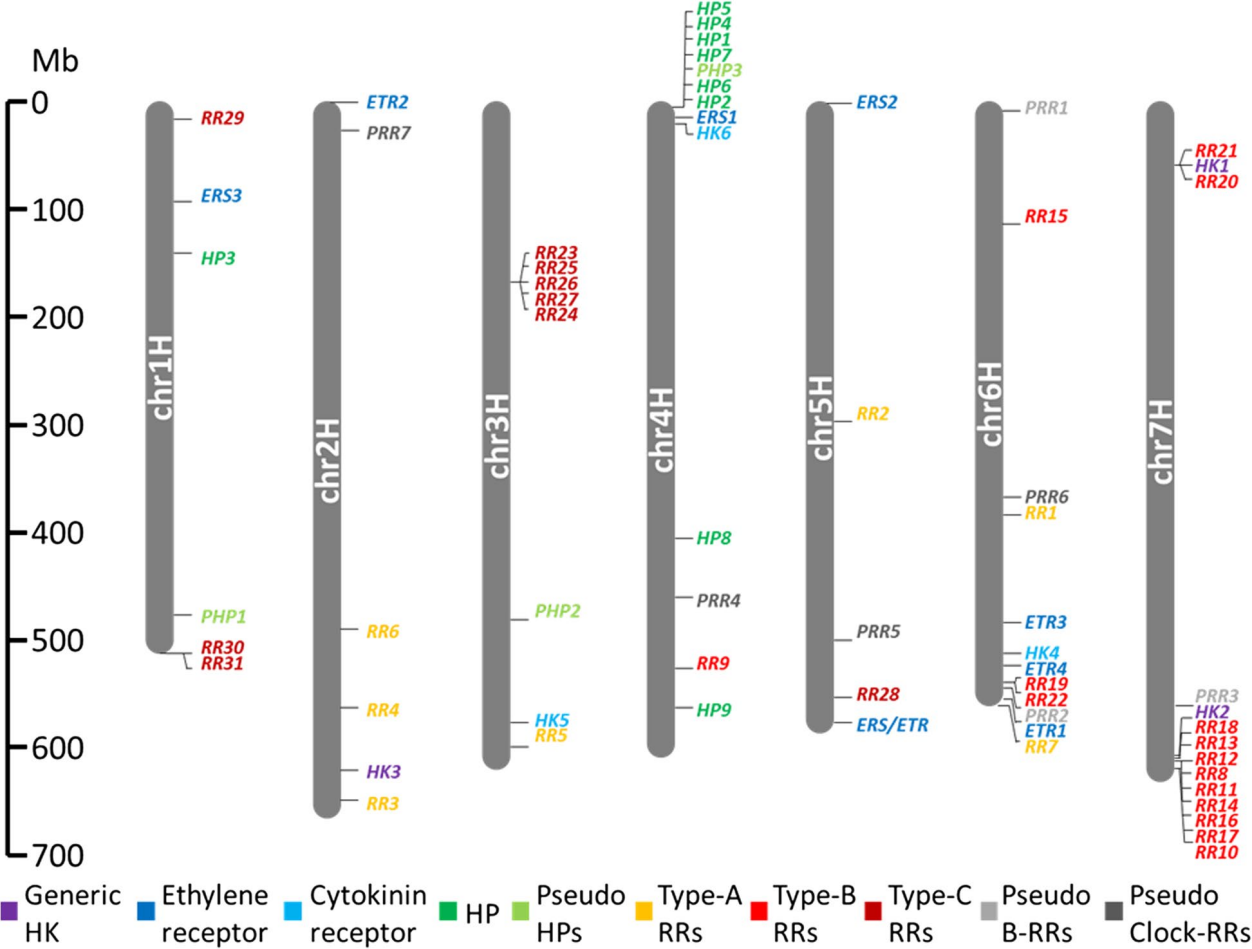
Chromosomal mapping of two-component signaling (TCS) elements in barley. Colors highlight subfamilies of TCS elements: purple: generic histidine kinases (HKs), dark blue: ethylene receptors, light blue: cytokinin receptors, dark green: histidine-containing phosphotransfer proteins (HPs), light green: pseudo HPs, orange: type-A response regulators (RR), light red: type-B RRs, dark red: type-C RRs, light grey: pseudo type-B RRs, dark grey: pseudo clock-RRs. The scale at the left of the chromosomal bar indicates the position on chromosomes (mega base pairs; Mb)

Sequence information on barley TCS genes is still fragmentary and mainly based on gene expression studies [14, 36, 37, 39] or older genome versions. We screened the latest release of the barley genome ‘Morex.v3’ [38] for genes encoding TCS components from all three main classes, only high-confidence (HC) genes were selected to avoid confounding effects of inaccurate gene models. In total, 67 non-redundant TCS elements, including receptor histidine kinases (HKs), histidine-containing phosphotransfer proteins (HPs) as well as different subgroups of response regulators (RRs) were identified, incorporating numerous previously unknown genes (Table 1, Table S1). Besides equal distribution of TCS genes across the seven chromosomes (Chr.), three loci in the genome containing highly similar gene copies from the same family stand out. These include clustered RRs from the type-C subgroup on Chr. 3H, clustered HP genes on 4H and clustered RRs from the type-B subgroup on 7H (Fig. 1). Close proximity and sequence similarity between the cluster genes indicates repeated events of tandem duplications which are predominantly found on the distal regions of the chromosomes.

To get information about gene representation in other cultivars, landraces and wild barleys, we screened the recently published barley pangenomes for presence of TCS genes [40]. Pangenomic data of 23 wild accessions, 36 landraces and 17 cultivars (Table S2; https://panbarlex.ipk-gatersleben.de) revealed that the majority of TCS elements is also present in most accessions/cultivars with few exceptions, such as HvHK3, HvRR3, HvRR33 or HvPRR2, that are not conserved in several pangenomes. Focusing on the three cluster regions on Chr. 3H, 4H and 7H, there is a tendency towards a reduced number of gene copies in wild accessions and landraces, particular for the HP cluster region. Results demonstrated that only three of the seven members of the HP cluster (*HvHP1*, *HvHP6* and *HvPHP3*) are commonly found in most of the accessions. Notably, the reference cultivar ‘Morex’ is the only accession that contained all cluster genes, exclusively HvHP5 which is not present in other modern cultivars. Orthologs of HvHP2, HvHP4 and HvHP7 are depleted in most of the accessions, but to a higher degree in wild barley and landraces. The same is true for type-B HvRR18 and type-C HvRR28, indicating that variation and accumulation of copy numbers at these distinct loci might be a consequence of domestication and selection for breeding.

**Table 1:**
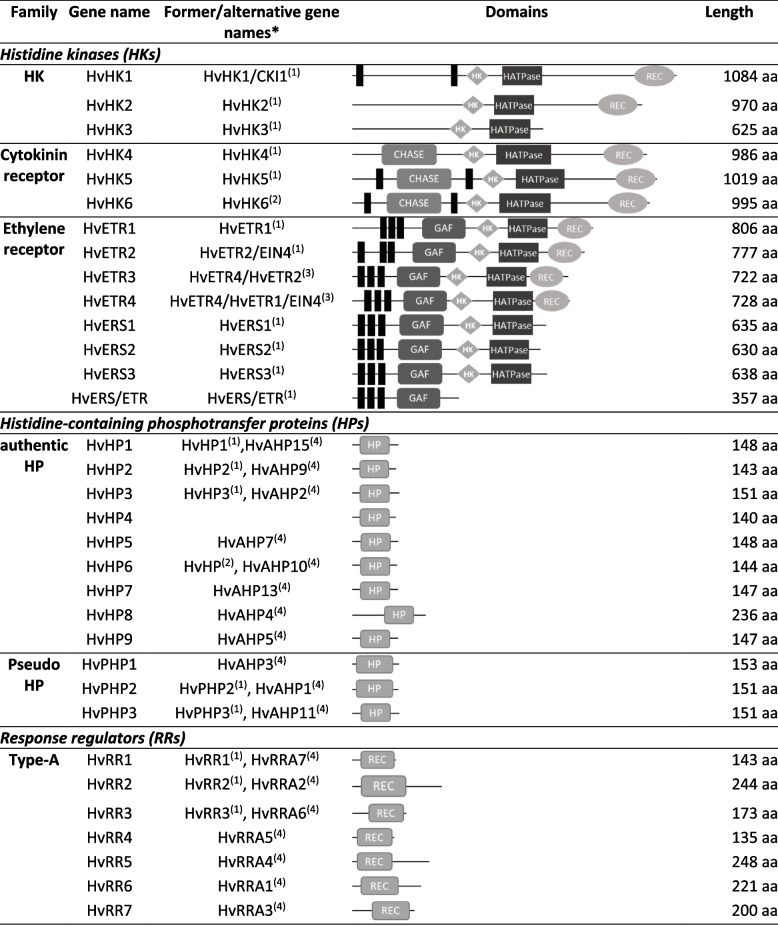

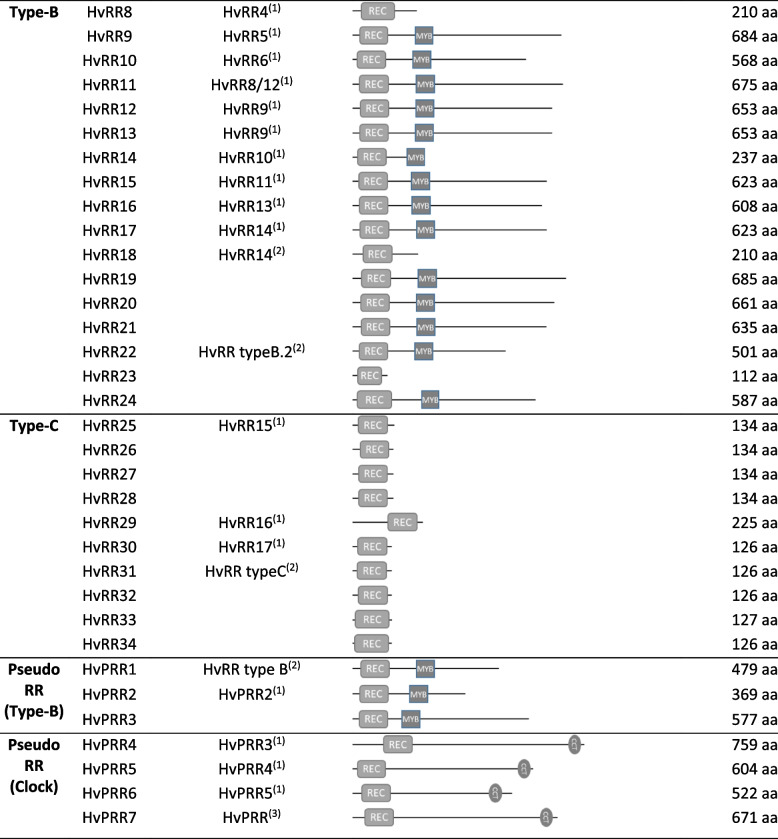
Elements of the two-component system (TCS) in barley are divided into different subgroups. Functional domains and protein length are shown in the table

Black boxes represent transmembrane domains; *gene names according to publications:(1) Thiel et al., 2012 [14]; (2) Hertig et al., 2020 [37]; (3) Hertig et al., 2023 [36]; (4) Radchuk et al., 2023 [39]

### Barley TCS genes and phylogenetic relationship with mono- and dicotyledonous species

The main components of multi-step phosphorelays (MSP) in plants are classified as histidine receptor kinases, His-containing phosphotransfer proteins and response regulators as output components of the system. To investigate phylogenetic relationships of phosphorelay elements, amino acid sequences of barley TCS elements were used for comparison with respective sequences from *Arabidopsis thaliana*, rice and maize (Table S3).

#### Histidine kinases (HKs)

**Fig. 2:**
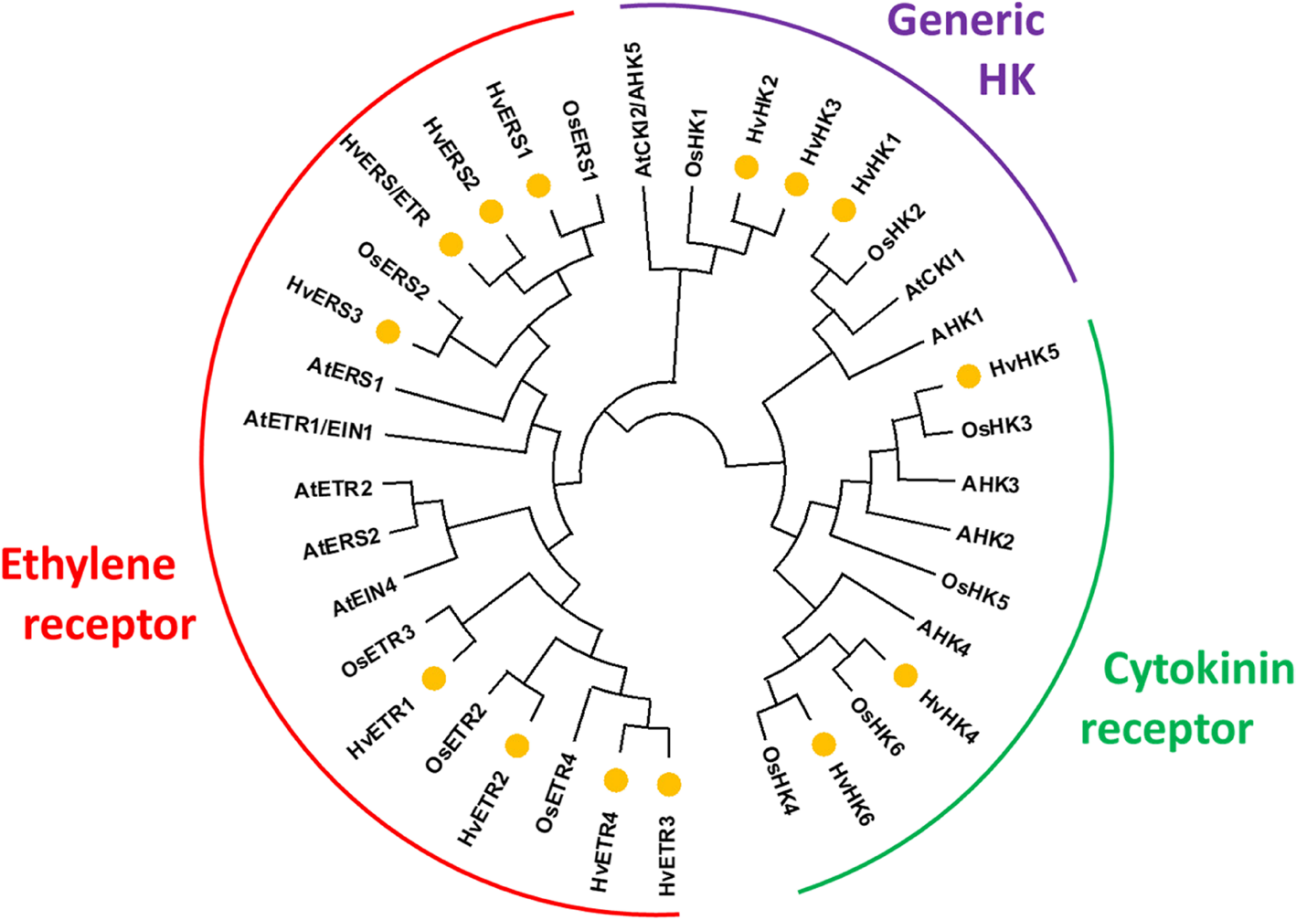
Phylogenic relationships of histidine kinases (HKs) from barley, *Arabidopsis* and rice. Barley proteins are marked with yellow circle; red: ethylene receptors; green: cytokinin receptors; purple: generic histidine kinases. Phylogenetic tree was designed with MEGAX

The class of hybrid HKs contains 14 genes, which can be separated into three subgroups (Fig. 2). (1) three generic histidine kinases (HvHK1, HvHK2, HvHK3) contain the histidine kinase (HK) domain, a HK-specific ATPase domain (HATPase) and a receiver (REC) domain. HvHK3 is lacking the REC domain at the C-terminal end. The HKs form two clades: HvHK1 clusters together with rice HK2 as well as *Arabidopsis* CKI1 and AHK1; whereas HvHK2 and HvHK3 have a higher similarity to rice HK1 and *Arabidopsis* CKI2/HK5. (2) HvHK4, HvHK5 and HvHK6 include an additional CHASE domain which is characteristic for cytokinin receptors. They are grouped with rice proteins: (a) HvHK4 with OsHK6, (b) HvHK5 with OsHK3 and (c) HvHK6 with OsHK4. Other rice (OHK5) and *Arabidopsis* (AHK2, AHK3 and AHK4) receptors are more distantly related to barley HKs. (3) the group of putative ethylene receptors consists of eight proteins: HvETR1 to HvETR4, HvERS1 to HvERS3 and HvERS/ETR which contain an additional GAF domain. The ERS factors lack the REC domain, HvERS/ETR contains only the GAF domain. Phylogenetic investigations showed a separation of ERS and ETR subgroups of barley close to rice orthologs: (a) HvERS1, HvERS2, HvERS3 and HvERS/ETR group together with OsERS1 and OsERS2; (b) HvETR1, HvETR2 as well as HvETR3 and HvETR4 form clades with OsETR proteins. The *Arabidopsis* ethylene receptors are more distantly related and did not show a clear separation between ERS and ETR elements.

#### Histidine-containing phosphotransfer proteins (HPs)

**Fig. 3:**
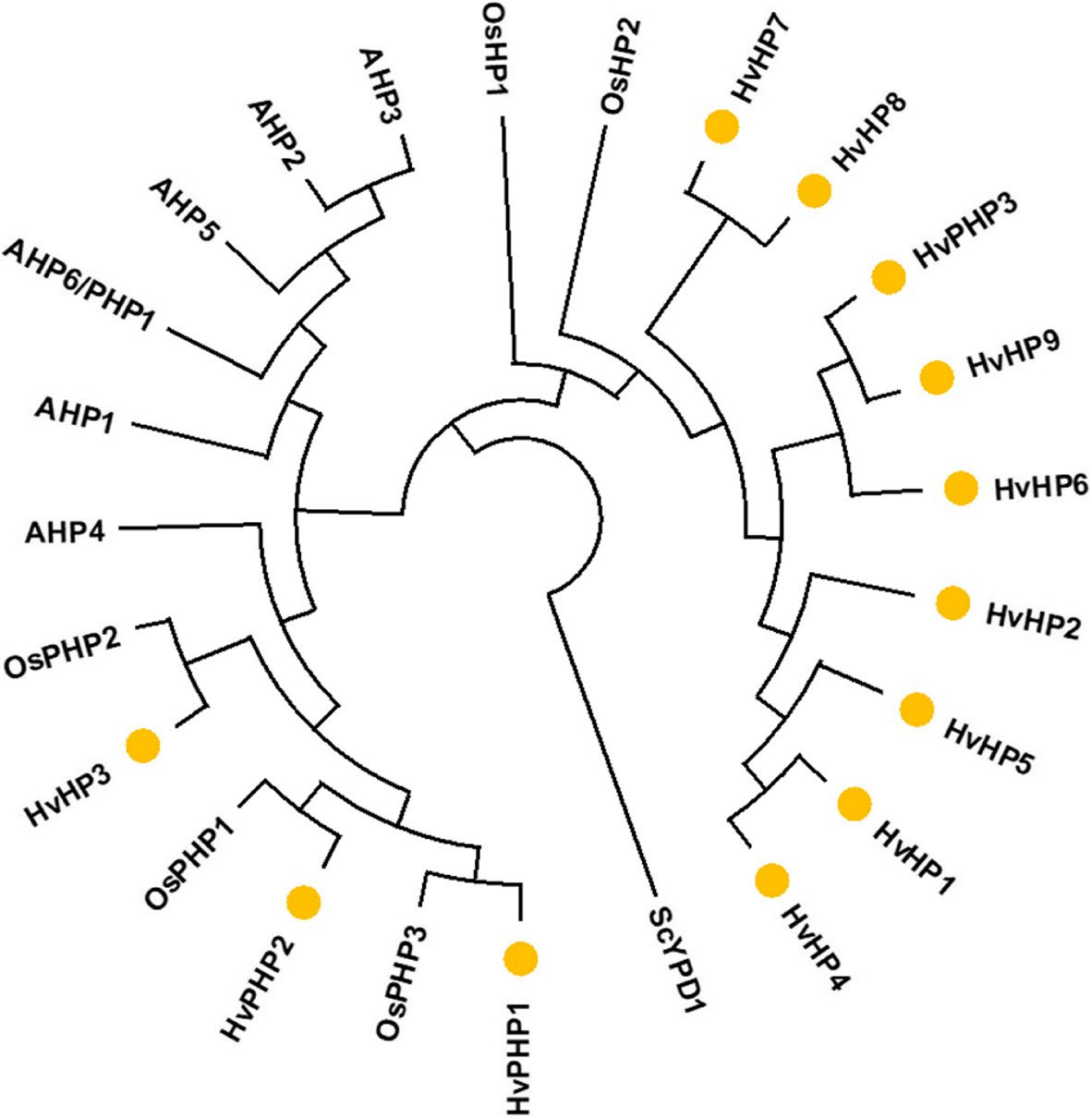
Phylogenic relationships of histidine-containing phosphotransfer proteins (HPs) from barley, *Arabidopsis* and rice. Barley proteins are marked with yellow circle, the yeast HP protein YPD1 was used as an outgroup. Phylogenetic tree was designed with MEGAX

The barley genome contains nine authentic HPs and three Pseudo HPs (PHP) where the active histidyl residue is replaced by Gln (Q) (Fig. S1a). Phylogenetic analysis separated two main branches of HPs. HvHP3, HvPHP1 and HvPHP2 group together with the rice PHPs1-3 while all *Arabidopsis* HPs formed an own dicotyledonous group. The other barley HPs (HvHP1, HvHP2, HvHP4-9 and HvPHP3) build a monocot-specific group together with both rice HPs, OsHP2 and OsHP1 (Fig. 3). In general, it is striking that the barley genome contains a significant higher copy number of HPs/PHPs compared to *Arabidopsis* (six) and rice (five), only the maize genome harbors a comparable number with nine HP genes [27]. The high number mainly originates from the HP cluster region on Chr. 4H revealing seven HP gene copies with high sequence similarity, particularly tandem genes in close proximity are nearly identical (e.g. HvHP1, HvHP4 and HvHP5 show more than 95% sequence identity to each other). We could not clearly distinguish HPs and PHPs due to high similarity.

#### Response regulators (RRs)

**Fig. 4:**
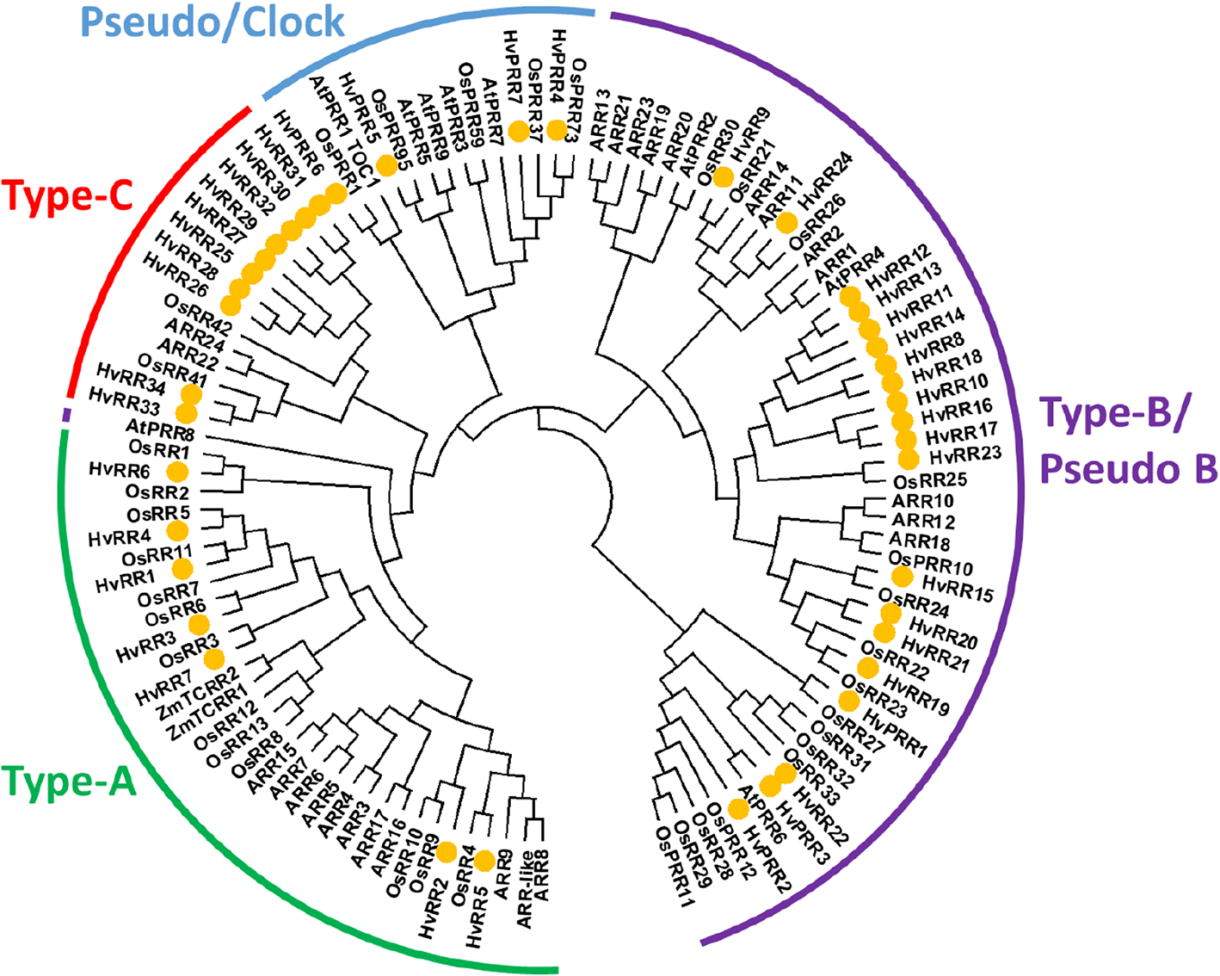
Phylogenic relationships of response regulators (RRs) from barley, *Arabidopsis*, rice. Maize elements assigned as transfer cell-specific (TCRR) are included in the analysis; barley elements are marked with yellow circle; green: type-A RRs; purple: type-B RRs and pseudo type-B RRs; red: type-C RRs; blue: pseudo clock-RRs. Phylogenetic tree was designed with MEGAX

The class of RRs can be separated into four types and five subgroups. Barley contains 34 authentic RRs that bear a specific Aspartate residue in the REC domain (Fig. S1b) and are grouped in three types (A-C) in the phylogenetic tree (Fig. 4). Type-A RRs include seven members (HvRR1 to HvRR7) containing only the REC domain, type-B members (HvRR8 to HvRR24) usually contain an additional MYB/SHAQHY DNA-binding domain, except of HvRR8, HvRR18 and HvRR23. The nine type B-RRs HvRR8, HvRR10-HvRR14 and HvRR16-HvRR18 are clustered on Chr. 7H (Fig. 1). Like the type-A RRs, type-C RRs (HvRR25-HvRR34) contain only a REC domain but are clearly separated from them. In the *Arabidopsis* and rice genomes only two type-C RRs exist whereas the number in barley is significantly higher probably due to tandem duplications similar to HP genes. The seven Pseudo response regulators (PRRs) lack the specific Aspartate residue within their REC domain (Fig. S1b). They can be divided into two subgroups: type-B Pseudo-RRs and Pseudo/Clock-RRs. Type-B PRRs contain an additional MYB domain and include HvPRR1-HvPRR3. The Pseudo/Clock-RRs including HvPRR4 to HvPRR7 bear an additional C-terminal CCT (CONSTANS, CO-like, and TOC1) domain.

Phylogenetic relationships between the barley RRs/PRRs with members from *Arabidopsis thaliana*, rice and maize revealed a higher similarity of barley elements with rice and maize in contrast to the dicot *Arabidopsis* (Fig. 4). Four main groups are visible: Type-A, type-B, type-C and Pseudo/Clock RRs. The type-A subfamily forms distinct individual groups with the rice genes illustrating the existence of rice and barley orthologues gene pairs. The *Arabidopsis* type-A RRs cluster separately and the maize transfer cell specific ZmTCRR1 and ZmTCRR2 [41] group with OsRR8 and OsRR12/OsRR13 in a distant relation to HvRR7. Clustered members of the type-B subfamily build a barley-specific branch with similarity to OsRR25. The other barley type-B RRs group together with the corresponding rice and *Arabidopsis* elements (Fig. 4), implying a higher conservation between these mono and dicot type-B elements. Barley PRRs, mainly the subgroup of clock genes, are closer related to individual members of *Arabidopsis* and rice PRRs. HvPRR7, also known as PPD-H1, functions as circadian clock gene controlling flowering time [42] and groups together with OsPRR37 and OsPRR73 showing no clear ortholog in *Arabidopsis*. Most of the *Arabidopsis* RRs form individual clades and ARR13, ARR21 or ARR23 are far away from all other type B RRs.

The analysis of the type C subgroup revealed 10 type-C RRs in the barley genome of which five proteins (HvRR25 to HvRR29) show a sequence identity of more than 97% to each other. They group together with HvRR30-32 and OsRR42, whereas HvRR33 and HvRR34 form an extra clade with OsRR41 and ARR22/ARR24.

### Expression profiles of barley TCS genes in vegetative and reproductive organs and tissues

**Fig. 5:**
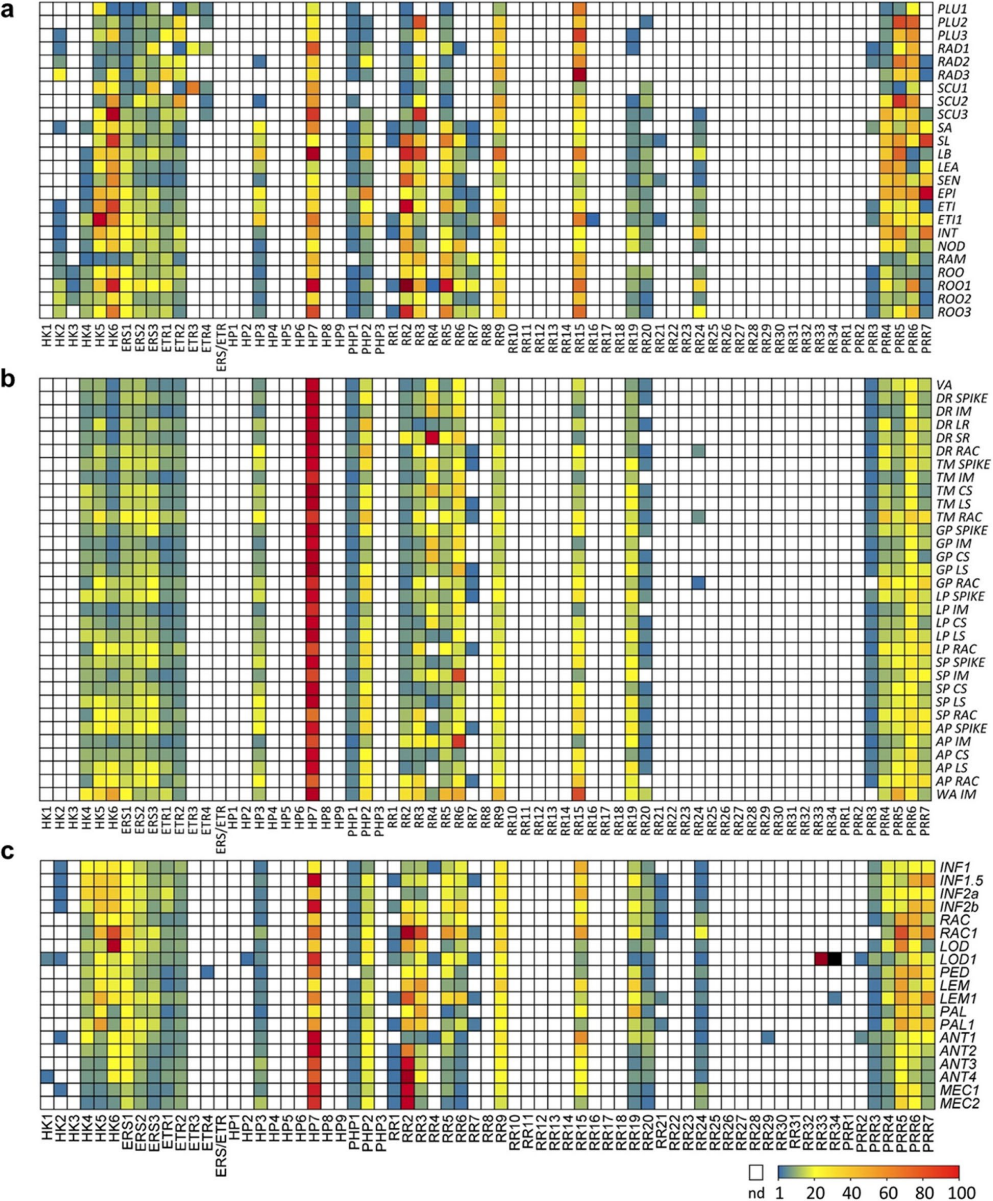
Heat map display of transcript levels of barley TCS genes in vegetative and reproductive tissues and organs. **a** vegetative tissues, **b** spike meristems/primordia and **c** floral organs. Expression levels (FPKM) from different RNA-seq datasets are displayed by color code. Blue- low, yellow- intermediate, red- high transcriptional levels and white boxes indicating not expressed (nd, FPKM < 1). *Abbreviations*: [a] PLU- plumule; RAD-radicle; SCU- scutellum; SA- shoot apex; SL- seedling; LB- leaf blade; LEA- leaf; SEN- senescing leaf; EPI- leaf epidermis; ETI- etioled leaf; INT- internode; NOD- node; RAM- root tip, ROO- root; [b] VA- vegetative apex; IM- inflorescence meristem; LR- leaf ridge; SR- spikelet ridge; CS- central spikelet; LS- lateral spikelet; RAC- rachis; stages: DR- double ridge; TM- triple mound; GP- glume primordia; LP- lemma primordia; SP- stamen primordia, AP- awn primordia; WA- white anther ; [c] INF- inflorescence; LOD- lodicules; LEM- lemma; PAL- palea, ANT- anther; MEC- meiocyte. Numbers indicate different time points or regions (see Table S4 for details)

Gene expression profiles give hints to gene functions and co-expression in a temporal and cellular context pinpoints to a coordinated participation in signaling modules. To capture global transcriptome profiles under stress conditions, in vegetative and reproductive organs, we used data from public repositories (BarleyExpDB database, http://www.barleyexp.com) [43–48]) to generate heatmaps of gene activities from barley TCS genes in different conditions (Fig. 5, Tables S5-S7, sample descriptions in Table S4).

#### Vegetative tissues

In germinating seeds, *HvETR3* and *HvETR4* are exclusively expressed in the scutellum, plumule and an early stage of radicle development (Fig. 5a, Table S5), coinciding with strong expression of *HvHP7* and some RRs (*HvRR3*, *HvRR9*, *HvRR15*, *HvPRR5*, *HvPRR6*). In leaves and leaf parts, a high expression of *HvHK5* and *HvHK6*, *HvHP7*, *HvPHP2* and various RRs (*HvRR2*, *HvRR3*, *HvRR5*, *HvRR9* and *HvRR15*) as well as all Pseudo/Clock *HvPRR5* and *HvPRR7* was observed, particular in the epidermis and the meristematic leaf blade. Roots of different ages showed expression of *HvHK6*, *HvERS1*, *HvHP7*, different RRs (*HvRR2*, *HvRR5* and *HvRR15*) and Pseudo/Clock RR *HvPRR6*.

#### Early development of spike meristems and primordia

We used data from a transcript atlas of LCM-isolated spike tissues that dissected central and lateral spikelets (CS & LS), inflorescence meristems (IM) and provascular tissues (RAC) at different premature stages of development [47] (Fig. 5b, Table S6). A strong expression of *HvHP7* and broad activity of putative cytokinin (*HvHK5/−6*) and ethylene receptors (*HvERS1-3*), *HvHP3*, *HvPHP2*, different RRs from the type-A (*HvRR3/−5*) and type-B (*HvRR9/−19*) as well as *HvPRR4* to *HvPRR7* could be detected in all tissues during development. However, some elements displayed dedicated expression in different stages of spike development. *HvRR4* showed a peak in the spikelet ridge of the double ridge stage (DR-SR) when reproductive development is initiated. *HvRR6* is highly upregulated in the IM where it even increased during progression to stamen primordia (SP) and awn primordia (AP) stages, whereas expression of *HvRR15* peaked in the IM in the late white anther (WA) stage. The most obvious tissue-specific expression was found for *HvRR24* in the rachis of the spike from the DR to glume primordia (GP) stage not expressed elsewhere in the spike (Fig. 5b).

#### Inflorescence and flower organs

A more generalized expression like in other tissues/organs could be seen for multiple TCS genes in floral organs between 30 and 55 DAF, but also some profiles with high specificity were recognized (Fig. 5c). *HvHK1*,* HvHK2*,* HvHP2* and *HvRR33/−34* are preferentially expressed in the lodicules (LOD1) at 55 DAF, *HvETR4* is exclusively expressed in the peduncle (but on a low level) and *HvRR29/HvPRR2* in young anthers. These genes are not transcribed in spike meristems, the precursor tissues from which inflorescence organs emerge, thus indicating that different phosphorelay modules are associated with phase transitions during floral development.

### Expression of barley TCS genes in grain compartments and specific cell-types during seed development

To get information about transcription of TCS genes in barley grain organs and tissues, we used published transcriptome data from whole grains and manually dissected grain compartments as well as a highly resolved cDNA library composed of LCM-dissected grain tissues for qPCR analysis. Generally, we observed largely different expression patterns in the grain with an array of genes that are not expressed in vegetative and floral organs/meristems.

#### Expression profiles of TCS elements in grain compartments

**Fig. 6:**
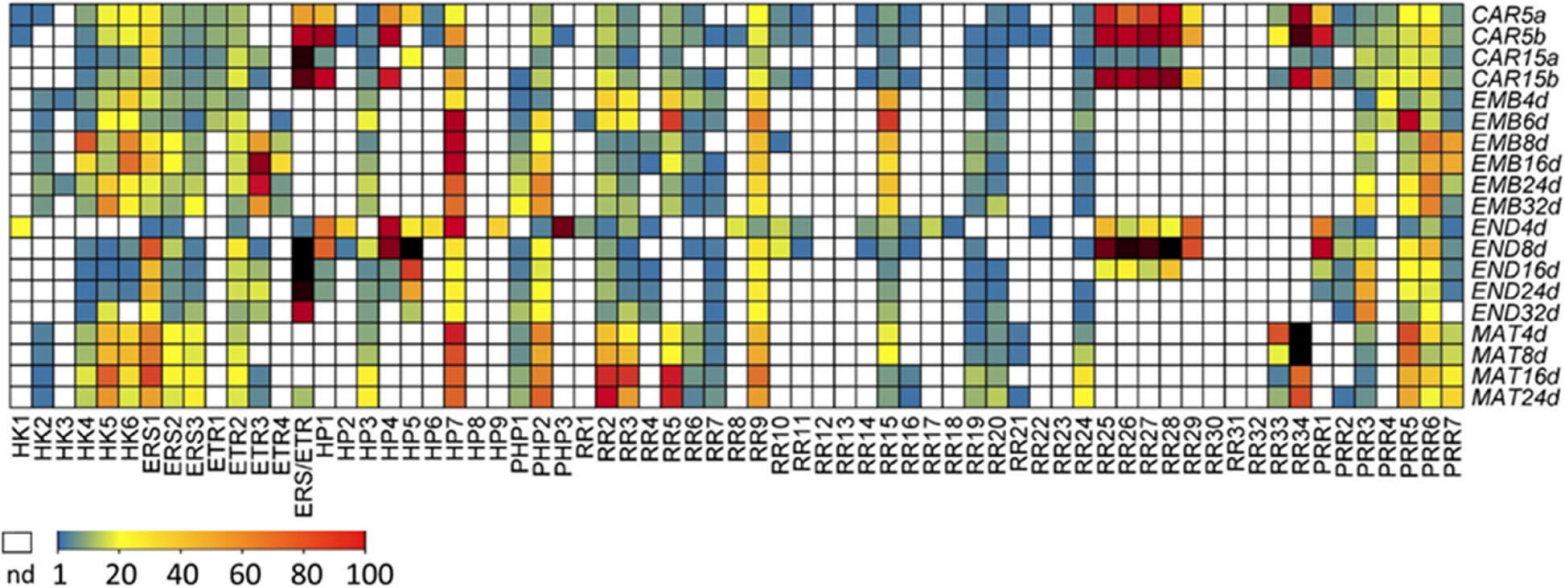
Heat map display of transcript levels of barley TCS genes in grains and grain compartments. Expression levels (TPM-/FPKM-values for the first six rows [CAR5a – EMB6d]) are displayed by color code. Blue- low, yellow- intermediate, red- high transcriptional levels and white boxes indicating not expressed (nd, TPM/FPKM < 1). Abbreviations : CAR- whole developing grain; EMB- embryo; END- endosperm, MAT- maternal seed tissues. Digits indicate days after flowering (DAF)

The gene expression atlas of developing barley seeds separating the three main grain compartments seed maternal tissues (SMATs, i.e. the pericarp and two-layered seed coat), endosperm and embryo [49] provides a higher spatial resolution compared to previous studies with whole grains or dissected embryo regions at 4 and 6 DAF (first six rows in Fig. 6). High expression of individual TCS elements monitored in whole grains [45, 46] can be attributed to distinct grain compartments and developmental stages (Fig. 6; Table S8).

In the endosperm fraction, the *HISTIDINE KINASE 1* (*HvHK1)*, a well-known master regulator of ETC cellularization [37], is specifically expressed during early development (4 DAF). Whereas the *HvERS/ETR* gene is outstandingly high expressed between 8 and 32 DAF coinciding with the beginning of the filling phase and starch accumulation in the endosperm Endosperm-specific expression was also observed for multiple HP genes (*HvHP1*, *HvHP2*, *HvHP4*, *HvHP5*, *HvHP6* and *HvPHP3)*, *HvRR1*, clustered type-B response regulators (*HvRR8*, *HvRR10/−11*, *HvRR14*, *HvRR17/−18*,* HvRR22)*; the type-C RRs (*HvRR25* to *HvRR29)* as well as *HvPRR1*, particular during early differentiation stages. A group of ERS genes is preferentially expressed in SMATs in conjunction with type-A RRs (*HvRR2/−3/−5*), *HvRR24* and *HvPRR5*. *HvRR33* and *HvRR34*, belonging to the C-type subgroup, showed a high specificity for the SMATs, not being expressed elsewhere (Fig. 6). A small number of genes including the histidine kinases *HvHK4*, *HvHK6*, *HvETR3*, and *HvRR15* are dominantly expressed in the embryo. Altogether, transcriptional profiles hint to compartment-specific activities and regulatory interactions in TCS signaling modules within the grain.

#### Expression of TCS genes in different cell types of the developing barley grain

**Fig. 7:**
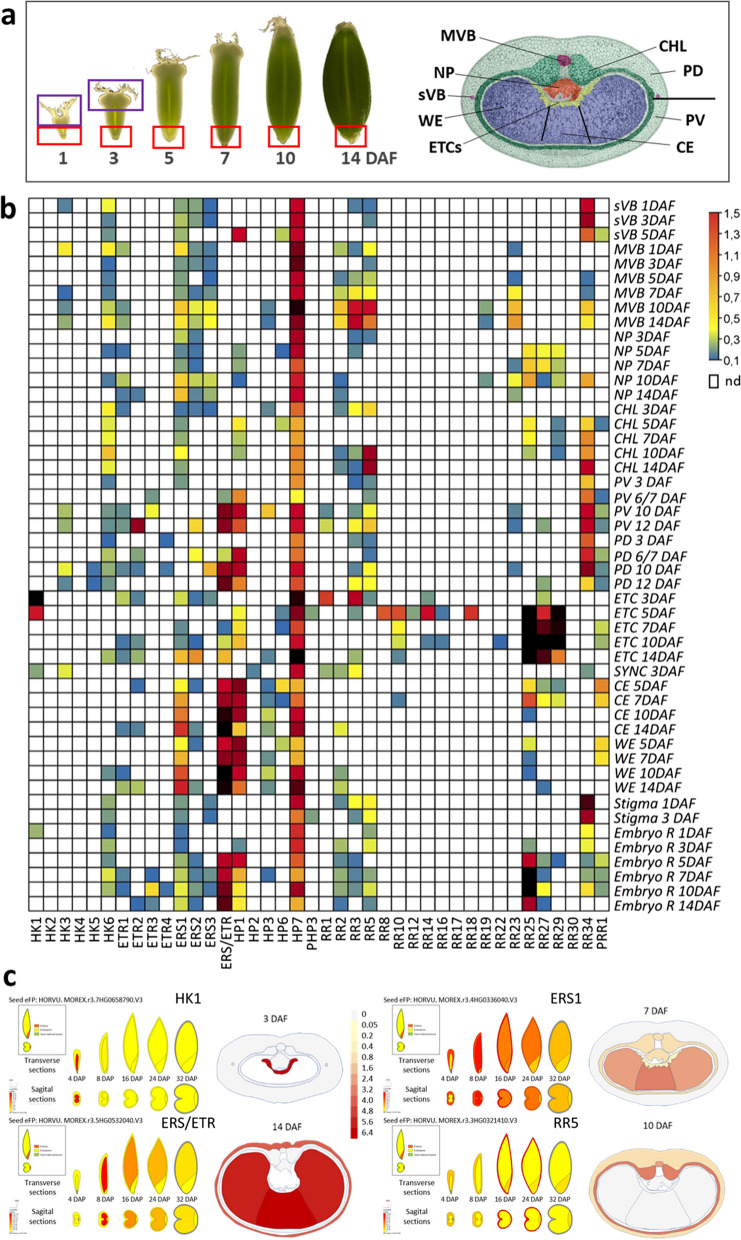
Relative expression of TCS genes in cell types/tissues of the barley grain across development. Samples of the cDNA library were isolated by LCM or manually dissected. **a** Representation of samples of the cDNA library. Left, grains at different developmental stages used for sampling, manually dissected regions are boxed, right, transverse section of a grain at 7 DAF, sampled tissue/cell types are labelled, horizontal axis of the grain is indicated by bar. **b** Heatmap of expression of selected TCS genes in grain tissues from 1 to 14 DAF. Transcript levels were determined by qPCR and given as relative expression. Color code illustrates expression levels: blue/green > 0.1 to < 0.3/low, yellow/orange > 0.3 to 1.0/intermediate, red > 1.0/high, white not detectable (nd, < 0.01). **c** Examples for refinement of RNA-seq data from grain compartments by qPCR and mapping of relative expression levels on seed cell types. Left, visualization of expression profiles of HK1, ERS1, ERS/ETR and RR5 in grain compartments by the Barley ePlant browser (https://bar.utoronto.ca/eplant_barley), transverse and sagittal sections show expression as TPM-values; right, visualization of relative expression levels of the genes in specific cell types/tissues of the grain at emblematic developmental stages, color code from white to red indicates expression levels. *Abbreviations*: sVB-side vascular bundle; MVB- main vascular bundle; NP-nucellar projection; CHL-chlorenchyma; PV-pericarp ventral; PD-pericarp dorsal; ETC-endosperm transfer cells; SYNC-syncytium; CE-central endosperm; WE-endosperm wings; Stigma-top region of the grain; Embryo R-embryo region of the grain

As the RNA-seq datasets are still on a lower level of resolution containing multiple cell types per sample, we used a cDNA library mainly composed of LCM-based isolated cell types of the grain to specify expression profiles by qPCR. The cDNA library contains 33 LCM-isolated samples with a focus on transport tissues within the grain, i.e. main vascular (MVB) and side vascular bundles (sVB); nucellar projection (NP) in maternal grain parts and the filial endosperm transfer cells (ETCs) (Fig. 7a, Methods S1). The seed maternal tissues (SMATs) were further divided in the dorsal and ventral pericarp (PD, PV) and the green chlorenchyma (CHL), and the filial endosperm tissues in the syncytial endosperm (SYNC, only 3 DAF), central endosperm (CE) and endosperm wings (WE). The developmental time frame covered the earliest stages of grain development until the start of grain filling (1 to 14 days after flowering (DAF)). Sample collection was complemented by manually dissected regions of the grain and grains (5 DAF) incubated with different hormones (Table S4).

Besides the ubiquitous expression of *HvHP7* in all cell types and domains of the grain, most of the genes displayed a high specificity concerning spatial and temporal activities. Among the histidine kinases, *HvHK6* is preferentially expressed in the CHL throughout all stages and partially in the MVB at very early (1 DAF) and late developmental stage (14 DAF). *HvETR2* showed a dedicated peak of transcriptional activity in PV at 12 DAF, *HvERS3* in PD at 10 DAF but also in MVB between 10 and 14 DAF (Fig. 7b). Other genes highly expressed in pericarp are *HvHP3* (PV at 10 DAF) and *HvERS/ETR*, *HvHP1* (PV/PD 6–12 DAF) or *HvRR34* (PV/PD 3–12 DAF) that also showed a maximum of expression in endosperm tissues (CE, WE) or in CHL (7–14 DAF), sVB between 1 and 5 DAF and stigma (1–3 DAF), respectively. The strong expression of *HvRR2*, *HvRR3* and *HvRR5* in SMATs (Fig. 6) was mainly originated from transcriptional abundancies in MVBs between 10 and 14 DAF and in case of *HvRR5* from enormous expression in CHL (10–14 DAF). A large portion of the TCS genes was found to be expressed in the filial endosperm. qPCR data unlocked that *HvERS1*,* HvERS/ETR*,* HvHP6* and *HvPRR1* are preferentially activated in central and endosperm wing regions (CE, WE). In contrast, genes such as *HvHK1*,* HvPHP3*,* HvRR1*, an array of type-B RRs (*HvRR8/−10/−12/−14/−16/−18*) and the type-C RRs (*HvRR25/−27/−29*) are more or less exclusively and strongly expressed in ETCs at various time points (Fig. 7b). *HvHK1* and *HvRR1* peaked during initial cellularization of ETCs (3 DAF), *HvPHP3* and type-B RRs after cellularization (5 DAF), and type-C RRs with extremely high expression levels during further differentiation of ETCs (7–14 DAF). The cDNA library of the syncytial endosperm (SYNC, 3 DAF) represents the multinucleate coenocyte, a peripheral layer surrounding the central vacuole, and can be regarded as a precursor for endosperm and aleurone cells [36]. Only few genes (*HvHK3*,* HvRR3)* are moderately expressed in SYNC in contrast to endosperm regions (CE, WE) at subsequent time points. Together, qPCR analysis enabled us to generate a high-resolution expression map of selected TCS genes narrowing down gene activities to distinct cell types of the barley grain (Fig. 7c; Table S9). We specified peaks of expression levels in maternal grain tissues, such as the pericarp, chlorenchyma and vascular tissues connecting the grains with the mother-plant, and filial grain compartments, like morphologically distinct parts of the endosperm, all of them important for grain development, storage product accumulation and final grain size.

#### Response of TCS genes to plant hormones

To analyze the individual response of TCS genes to plant hormones, grains (5 DAF) were incubated in media supplemented with ABA (50 µM), synthetic auxin 1-Naphthaleneacetic acid (NAA, 50 µM), cytokinin 6-Benzylaminopurine (BAP, 5 µM) and the ethylene precursor 1-Aminocyclopropane-1-carboxylic acid (ACC, 10 µM) for 16 h. qPCR analysis showed that all hormones induced altered expression levels of candidate genes, either up- or downregulated (Fig. S2, Table S10). *HvHK1*,* HvHK4*,* HvHP6*,* HvRR1*,* HvRR8*,* HvRR30* and *HvPRR1* are significantly upregulated by ABA, whereas the *HvRR2* and *HvRR23 genes* are downregulated. Besides *HvHK1*, putative ethylene receptors *HvETR1* and *HvETR4*,* HvRR8*,* HvRR22* and *HvPRR1* are activated by ACC. Response to NAA and BAP is broadly similar, but differs to the other hormones: *HvHK3*,* HvHK6*,* HvRR2*,* HvRR5*,* HvRR27*,* HvRR30* and *HvRR34* are commonly downregulated, in contrast to stimulated expression of few genes, such as *HvHK1*,* HvRR1* and *HvRR22* by BAP or *HvHK1*,* HvRR8*,* HvRR14* and *HvRR29* by NAA. Studies indicated different hormonal influences and crosstalks with the transcriptional regulation of TCS signaling elements in barley grains.

### *TCSn::GFP* activity in barley grains

Type-B RRs contain an additional MYB domain and act as transcription factors that regulate the expression of downstream target genes. Since a subgroup of barley type-B RRs is specifically expressed in the ETC region at 5 DAF, we used the *TCSn::GFP* reporter in barley to monitor the type-B activity *in planta*. The artificial TCSn promoter consists of 12 repeats with variations of *Arabidopsis* type-B RR DNA-binding motifs and was shown to monitor the phosphorelay signaling output in diverse plant organs and tissues [50]. TCSn-based reporters have also been used in monocot species, like maize, rice and barley [50–52]. Relying on functional conservation in monocots, barley plants from two homozygous transgenic lines (E4 + E7) were investigated for the presence of GFP signals by confocal microscopy.

**Fig. 8:**
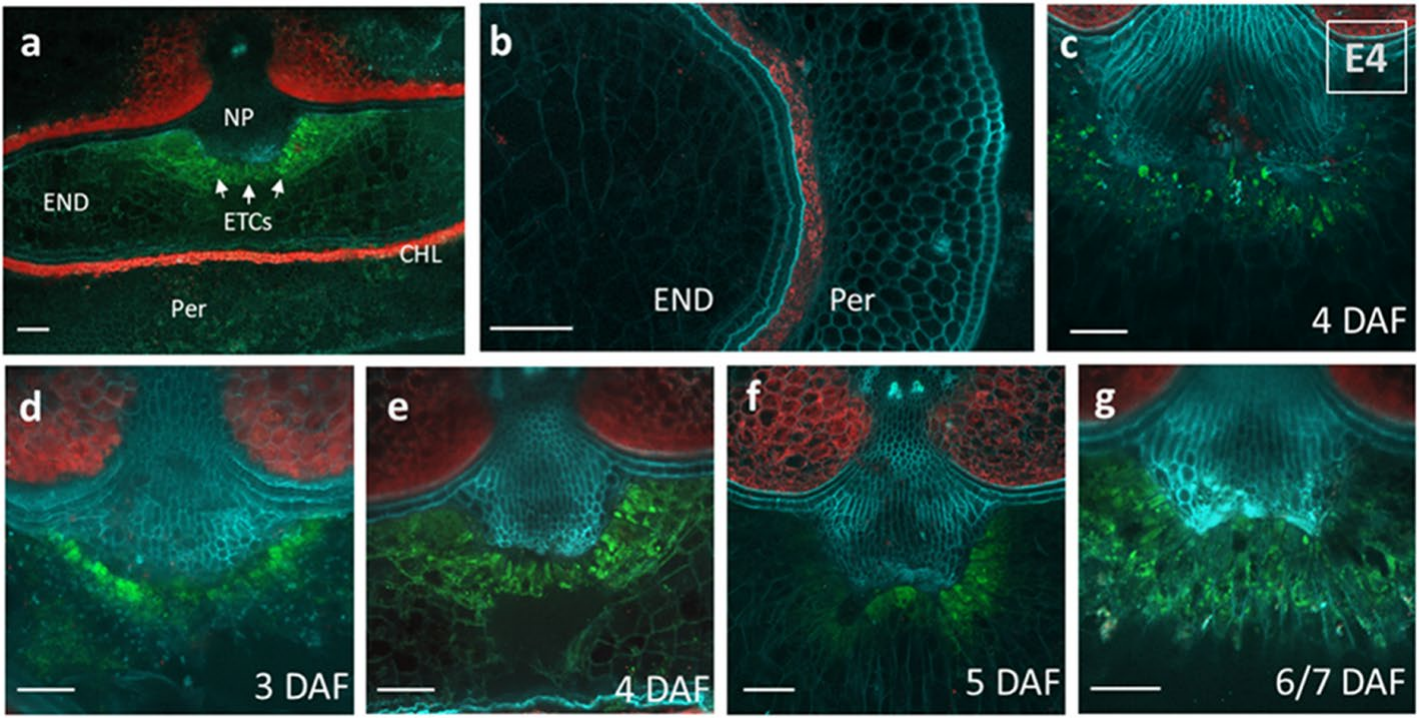
* TCSn::GFP* activity in developing barley grains. Two independent transgenic lines were examined and exhibited similar reporter gene activities (line E7 shown as representative). **a** transverse section of the central part of the grain at 4 DAF shows strong fluorescence in the ETC region (white arrows). **b** Magnification of the endosperm wing region and the pericarp at 5 DAF; (**c**-**g**) magnification of the ETC region at the maternal-filial boundary of grains. c grain from line E4; d - g grains at successive time points during endosperm differentiation. CHL-chlorenchyma; ETC-endosperm transfer cells; END-endosperm; NP-nucellar projection; Per-pericarp; bars = 100 μm

No GFP signals were found in any of the analyzed vegetative tissues, i.e. roots from early seedlings (5 DAG), leaf blade or stem internodes (30 DAG). In contrast, strong and dedicated signals could be detected in the ETC region of developing grains (Fig. 8a). Starting with the onset of endosperm cellularization at 3 DAF, outstandingly high GFP activity was exclusively present in the first layer of dividing ETCs (Fig. 8d). Fluorescence emerged to the three ETC-layers during further differentiation (Fig. 8e), whereas with the beginning of the storage phase around 10 DAF fluorescence signals disappeared. No GFP activity was observed in the endosperm and maternal grain tissues (pericarp) (Fig. 8b) underlying ETC-specificity of the signaling output, which was also confirmed by using grains from transgenic line E4 (Fig. 8c). Treatment of plants with the synthetic hormones BAP (cytokinin) and NAA (auxin), did not significantly induce ectopic GFP activity in other grain tissues (Fig. S3). Collectively, reporter gene output substantiated high specificity of type-B RR activity in barley ETCs that largely correlates with expression profiles from tissue-specific analyses (Fig. 7b).

### Response of barley TCS genes to stress

To get information about responses to abiotic and biotic stresses, we extracted expression data of TCS genes from experiments involving heat [53], drought [54] and cold stress [55], light and dark treatment [43] as well as biotic stress by infection with *Fusarium* [56]. Only a subset of TCS elements showed significant differences in transcript levels upon treatments (Fig. S4, Table S11). *HvRR2* is upregulated after drought treatment in young inflorescences of wild barley whereas *HvRR2*,* HvPHP1* and *HvPRR5* are downregulated in leaves of the cultivar ‘Scarlett’. *HvHK6*,* HvRR2* and *HvPRR6* responded to heat stress in roots, *HvPHP1*,* HvRR2*,* HvRR3*,* HvPRR4-HvPRR7* depicted a changed expression after cold treatment. These genes, together with *HvRR3*,* HvRR5* and the type-B RRs *HvRR9/−15/−24*, are also differentially expressed after light/dark treatment. Infection with *Fusarium* revealed a time- and strain-dependent reaction of *HvHK5/−6*,* HvHP1/−4*,* HvRR2*,* HvRR25-HvRR28* and *HvPRR1* mostly being upregulated after 4d treatment with strain 3ADON (Fig. S4). Together, we did not observe a broad response of TCS genes to the different kind of stresses, instead, individual TCS elements seem to be commonly involved in stress responses.

## Discussion

This study provides a complete compilation of the TCS genes in barley and resulted in a total number of 67 non-redundant sequences in the latest barley genome (Morex.V3). Chromosomal localization, phylogenetic relationships to known elements of *Arabidopsis* and rice and comprehensive gene expression profiles in barley plants pinpoint to diverse functions in organs and tissues/distinct cell types throughout the plant development.

### The high number of TCS genes in barley might be a result of domestication

The number of TCS elements is higher than in the model species *Arabidopsis*, rice and maize with 51, 52 or 50 genes (without phytochrom receptors), respectively, and most of all reported species. Only few polyploidic species, like *Brassica rapa* or *Glycine max*, contain more TCS sequences. Phylogenetic analysis shows that the barley genes are more closely related to the rice (and maize) ones and often build monocot-specific branches revealing that expansion of TCS genes in barley occurred after the divergence of monocot and dicot plants. The increased number of genes compared to other members of the Poaceae family, rice and maize, seems to be due to the occurrence of clustered genes at distinct chromosomal loci (Chr. 3H- type-C RRs, Chr. 4H- HPs and Chr. 7H- type-B RRs). These loci are predominantly found on the distal regions of the chromosomes known to bear increased gene density compared to proximal parts which is in congruence with characteristics for genetic diversity and recombination frequency. The accumulation of duplicated genes in close proximity at distinct chromosomal regions in modern barley cultivars might be hotspots for selection during the domestication process. This is supported by new insights from pangenomic data [40] which show the tendency that in wild barley or landraces the amount of cluster genes, particular for HP genes, is reduced (see example Fig. S5). Nearly identical gene copies and/or high sequence identity of cluster genes indicate recent duplications in terms of selective evolution. As long-term breeding efforts were mainly targeted for large and round grains and grain weight [57], these loci with gene copies might have beneficial effects on grain formation and finally, grain yield. Our data show that most of the cluster genes bear a cell type-specific expression in filial grain parts, explicitly in ETCs, thereby implying that enhanced expression is an important driver for the evolution of modern cultivars.

### Expression profiles of TCS genes pinpoint to diverse functions in plant’s life

Expression profiles of TCS genes show specific activities in plant parts, organs and tissue compartments at different developmental time points which suggest coordinated participation in locally restricted signaling modules. We could identify individual HKs, HP intermediates and RRs from all subgroups that are transcriptionally activated in vegetative tissues and reproductive organs. For example, *HvHK5*, *HvHK6*, *HvHP7* and *HvRR2/−5/15* are expressed in leaf parts and roots whereas putative ethylene receptors *HvETR3/−4* are preferentially expressed in early seedling organs. In developing spike meristems, distinct RRs (*HvRR4/−6/−15/−24*) are specifically expressed in primordia of spikes, such as the spikelet ridge, inflorescence meristem or rachis, and might be regulators of cell fate decisions and meristematic activity within in the spike, probably influencing grain-set and number in barley. A subset of TCS elements, like *HvHK5/−6*,* HvPHP1*,* HvRR2* and *HvPRR5*, was shown to commonly respond to different kinds of abiotic and biotic stresses, and might be interesting targets for future breeding efforts to improve adaptation and resistance against unfavorable conditions as exemplified in rice. Knockdown of *OsRR26* and mutagenesis of *OsRR22* improved salinity tolerance in young seedlings [58, 59]; OsHK1 regulates root growth under semi-aquatic conditions by mediating ethylene signaling pathways in interaction with OsERS2 [12].

### Grain specificity might be linked with grain size and yield

The most obvious observation from expression studies was the grain-specificity of certain TCS genes implying regulatory interactions within the grain. Among the HKs, *HvHK1*,* HvERS/ETR* and *HvETR3/−4*, an array of HP proteins (with the exception of HvHP3 and HvHP7) as well as the type-B RRs (*HvRR8*,* HvRR10/−11*,* HvRR14*,* HvRR16-18*,* HvRR22*) and type-C elements (*HvRR25-29*,* HvRR33/−34*) are exclusively expressed in the grain compartments embryo, endosperm and maternal tissues. Information about the function of TCS signaling modules in seed development of plants is rare with few exceptions. *Arabidopsis* HISTIDINE KINASE 1 (AHK1) was initially identified as plant osmosensor [60], but was also shown to be a positive regulator of ABA signaling which impacts seed maturation [13]. Its close homolog CYTOKININ INDEPENDENT1 (CKI1) is required for female gametophyte development [61] and specifies the central cells, the gametic precursor of the endosperm [62]. In rice, the putative ethylene receptor ETR2 has an impact on grain filling and grain weight by modulating ethylene sensitivity [63].

We specified expression profiles from RNA-seq data by qPCR analysis and attributed transcriptional activities to different cell types within maternal and filial grain compartments. The histidine kinases *HvHK3*,* HvETR2-4*,* HvERS3* and *HvHP3* are more or less specifically expressed in the pericarp which is important for early grain development as it determines grain length and finally grain shape and size [64]. The pericarp is interspersed by the green chlorenchyma (CHL) layers adjacent to the endosperm, the MVB and NP in the crease region and small vascular bundles at the horizontal axis of grains (sVB) which degenerate around 5 DAF. A particular expression in the CHL was observed for *HvRR5*, whereas other type-A RRs (*HvRR2/−3*) and type-B *HvRR23* are preferentially expressed in MVB and/or *HvRR34* shows a peak of transcriptional activation in sVBs during early development (1–3 DAF), particular in the apical grain region. Embedded in the pericarp, MVB, NP and sVBs represent the transport route for assimilates from the mother plant into the grain which controls assimilate partitioning and supply to the endosperm [65]. Subsequently, an effect of nutrient transport capacity on development of maternal and filial grain parts and the important trait grain size can be concluded. Barley RRs might play a similar role in grain’s vascular differentiation as demonstrated for CKI1, AHK2/−3 and ARRs that were shown to regulate proliferation and differentiation of cell lineages in vascular strands of *Arabidopsis* inflorescence stems and roots [20, 21].

The majority of the grain-specific TCS genes is expressed in filial endosperm tissues: putative ethylene receptors *HvERS1*,* HvERS/ETR*,* HvHP1/−3/−6* and *HvPRR1* are dominantly expressed in the central and wing regions of the endosperm coinciding with cellular differentiation and the onset of the storage period. ETCs at various developmental time points depicted specific expression of multiple TCS genes. *HvHK1*,* HvPHP3*,* HvRR1* as well as type-B (*HvRR8* to *HvRR16*) and type-C RRs (*HvRR25/−27/−29*) are outstandingly high and exclusively expressed in ETCs at key stages of differentiation. A stringent ETC-specificity of these genes was previously shown by pyrosequencing of developing barley ETCs [14] which proposed a function in cell specification and further differentiation of ETCs. In line with that is another LCM-based transcriptome study that dissected subdomains of the syncytial barley endosperm and identified numerous TCS elements as highly enriched in ETCs [36]. In maize, *ZmTCRR-1* and *− 2* were found to be specifically expressed in transfer cells of kernels [41], but functional data for kernel development are missing. The only functional study in a cereal species showed that HvHK1 is a central regulator of transfer cell identity in the young barley endosperm [37]. Knock-down of *HvHK1* leads to disturbed ETC specification, impaired differentiation of adjacent endosperm tissues and smaller grains. Gene expression analysis and prediction of gene regulatory networks revealed that TCS phosphorelays directed by HvHK1 are main regulatory links for the initial steps of endosperm cellularization. Intriguingly, several of these genes belong to the gene cluster on Chr. 7H supporting the idea that gene duplications impact grain yield in barley. Besides specific expression in ETCs, the role of type-B RR genes as transcriptional activator for ETC differentiation is validated by *TCSn::GFP* activity in living barley grains. The TCSn-reporter was used for the first time in seeds of an important crop species to monitor type-B RR activity and its transcriptional output. A strong and unique activity is obvious in ETCs at decisive stages of differentiation: fluorescence signals are visible at the first steps of endosperm cellularization and spread to all three layers of ETCs until the beginning of the storage phase (10 DAF), the time point when ETCs attain full functionality in nutrient transfer [35]. The sharp decrease in activity after 10 DAF overlaps with transcriptional activity of type-B RRs and illustrates that these genes are able to activate downstream target genes in ETCs of barley grains. Results from Kirschner and colleagues [52] using the *TCSn::Venus-H2B* construct in barley showed *TCSn*-activity in the root cap and stele which is barely detectable and could be enhanced by application of synthetic cytokinins. We could not monitor *TCSn*-activity in vegetative tissues in our analysis, this might be due differences in experimental conditions, the reporter gene and/or plant material. Together, strong and locally restricted activity under native conditions underlines the dedicated signaling output in ETCs during differentiation.

*In conclusion*, this work presents a comprehensive overview on TCS genes in the temperate cereal crop barley. The genes could be allocated to all known subfamilies of the phosphorelay and showed an unexpected abundancy in barley compared to other cereal crop species. Increased number of gene copies is mainly originated by tandem duplications forming complex loci in the genome that might be hotspots of selection during the evolution of modern cultivars. These genes are exclusively expressed in filial grain tissues, predominantly the ETC region, and are deemed to affect cellular differentiation in the early endosperm and grain filling at later stages. Expression of TCS elements in vegetative and reproductive organs/tissues and response to stresses revealed different activated signaling modules potentially affecting diverse agronomic traits. Identification of beneficial alleles is of importance for breeding of superior varieties and application of novel technologies, like gene editing, to improve yield potential in the future. Data will be also valuable for studies with other grain crop cereals, such as wheat, maize or rice.

## Methods

### Identification of barley TCS genes isolation and phylogenetic analysis

To identify all members of the two-component system (TCS) signaling pathway in barley, we used the BLAST platform of BARLEX (https://apex.ipk-gatersleben.de/apex/f?p=284:10) and full-length gene sequences of previously described TCS genes [14]. We compared the presence of genes available in the Morex reference genome v1 [45] with the more recent Morex reference genomes v2 [66] and v3 [38]. High confidence (HC) genes were selected and checked for the presence of functional domains by using the CDD v3.20 and Pfam v34.0 databases of the Conserved Domain Database of NCBI [67] (https://www.ncbi.nlm.nih.gov/Structure/cdd/wrpsb.cgi). Barley TCS genes from the different subgroups were annotated according to the *Arabidopsis* and rice nomenclature and numbered subsequently, former gene assignments used in other publications are given in Table 1. Sequences with more than 90% identity were considered as duplicated genes. Chromosome distribution was visualized using Gene Location Visualize from GTF/GFF from TBtools v1.119 software [68]. Phylogenetic studies of barley TCS peptide sequences were conducted using MEGA X software [69], which included peptide sequences from *Arabidopsis thaliana* [23], *Oryza sativa* [24] and *Zea mays* [27, 41, 70]. Multiple protein sequence alignments were performed using MUSCLE [71]. The evolutionary history was inferred by using the Maximum Likelihood method and JTT matrix-based model [72].

### Determination of expression values from RNA-seq databases

Expression values of barley TCS genes given as fragments per kilobase and million (FPKM values) were extracted from public RNA-seq datasets using BarleyExpDB: The Barley Expression Database [43] (http://barleyexp.com/). Following datasets were used: SRA BioProjects PRJNA496380 [44], PRJNA755156 [46], PRJEB39672 [47], PRJEB14349 [45], PRJNA558196 [48], PRJEB18276 [55], PRJNA728113 [56], PRJNA324116 [53], PRJEB12540 [54], PRJNA781996 and PRJEB21096. Data from grain compartments were obtained from [49] and are given as transcript per million (TPM) values. FPKM/TPM values below 1 were considered as non-detectable. Barley sequence information is publicly available in the Barlex genome explorer (https://apex.ipk-gatersleben.de/apex/f?p=284:10). Heatmaps were designed by using the Heatmap Illustrator of TBtools v1.119 software [68].

### Quantitative PCR studies

The expression of selected barley TCS genes was investigated using a tissue-specific cDNA library generated at IPK Gatersleben which allows expression profiling of any gene of interest (detailed information in Methods S1). The library is composed of LCM-isolated grain tissues at different developmental stages and manually dissected grain organs, as well as hormone-treated grains. Sample processing has been performed as described in [73]. Briefly, harvested grains were immediately frozen in liquid nitrogen and glued onto sample plates by O.C.T medium. Serial cross sections (20 μm) were mounted on PEN membrane slides (PALM, Carl Zeiss Micro Imaging GmbH, Jena, Germany), dried and tissue sections were isolated and collected using the PALM^®^ MicroBeam laser system (PALM). Usually between 30 and 300 tissue sections from at least 5 individual grains were pooled for one sample. RNA was extracted using the Absolutely RNA Nanoprep Kit (Agilent) and total RNA was amplified by one round of T7-based mRNA amplification using the MessageAmp aRNA Kit (Invitrogen) to generate antisense RNA (aRNA). After quality assessment, 300 ng of aRNA was processed for first strand cDNA synthesis using the SuperScript^®^ III First-Strand Synthesis System for RT-PCR (Invitrogen, Carlsbad, USA) to generate normalized cDNA libraries. The Power SYBR Green PCR Mastermix was used to perform reactions with three replicates per sample in an ABI 7900 HT Real-Time PCR system (Applied Biosystems, CA, USA, https://www.thermofisher.com). The data were analyzed using SDS v.2.3 software (Applied Biosystems), and PCR efficiencies (E) were determined by LinRegPCR software (https://www.gene-quantification.de/download.html#linregpcr). The relative expression (1 + E)^−ΔCT^ normalized to the expression of *HvActin* (HORVU.MOREX.r3.5HG0457850.1) was used to calculate transcript levels according to [74]. Expression levels below 0.1 were considered as non-detectable. To analyze the response of TCS genes to plant hormones, harvested grains at 5DAF were incubated in 1/2 MS media for 16 h either supplemented with abscisic acid (ABA, 50 µM), the synthetic auxin 1-Naphthaleneacetic acid (NAA, 50 µM), cytokinin 6-Benzylaminopurine (BAP, 5 µM), the ethylene precursor 1-Aminocyclopropane-1-carboxylic acid (ACC, 10 µM) or water for mock treatment. To assess the effect of hormone treatment, ΔΔCt-values were calculated representing the log_2_-ratio compared to control conditions (mock treatment). The primers used are listed in Table S12.

### Visualization of relative expression in grain tissues by thematic maps

Microsoft Excel features were used to draw custom thematic maps of barley grains for plotting of expression data [75]. To this end, a macro for the automatic depiction of the expression data onto the graphical thematic map was created. The drawing of the grains and cell types/tissues is based upon [76]. In addition, a scalebar for the heatmap was used that allows to color-code the different tissues according to the expression levels of the genes.

### Generation of *TCSn::GFP* barley lines

The *TCSn::GFP* expression unit [50] was transferred into the binary vector p6i-d35S-TE9 (DNA Cloning Service, Hamburg, Germany) for barley transformation. Transgenic lines were generated by *Agrobacterium tumefaciens*-mediated transformation of immature embryos from *Hordeum vulgare* cv. Golden Promise as described [77]. The regenerated plants were investigated for the presence of the selection marker *hygromycinphosphotransferase* (*hpt*) by PCR, homozygous plants were selected and two lines (E4 + E7) were investigated in the T3-generation.

### Microscopic investigation of *TCSn::GFP* barley lines

Transgenic barley plants were grown in greenhouses at 18 °C with 16 h light and 60% air humidity and flowers were tagged as described [78] to estimate grain developmental stages. Spikes between 3 and 10 DAF were cut at the base and incubated in 1/2 MS media overnight (16 h), for hormone treatment NAA (20 µM) or BAP (5 µM) was supplemented to the media. Transverse median sections of harvested grains were examined using a Zeiss CLSM780 laser scanning microscope (Carl Zeiss, Jena, Germany). GFP was excited with a 488 nm laser line and emission analyzed with a 490–540 nm bandpass. Signal specificity was confirmed by photospectrometric fingerprinting using lambda signature.

## Supplementary Information

Additional file 1: Figure S1. Phosphorylation sites in authentic and pseudo HPs and RRs. (A) Histidyl phosphorylation site in HPs (red) and alternative amino acid in PHPs (blue). (B) Aspartate phosphorylation site in RRs (red) and alternative amino acids in PRRs (blue). Figure S2. Altered expression of barley TCS elements after treatment with hormones. Grains (5DAF) were incubated for 16 h either with abscisic acid (ABA, 50 µM), synthetic auxin 1-Naphthaleneacetic acid (NAA, 50 µM), cytokinin 6-Benzylaminopurine (BAP, 5 µM) or the ethylene precursor 1-Aminocyclopropane-1-carboxylic acid (ACC, 10 µM). Color code illustrates ΔΔCt values corresponding to log_2_-fold change (hormone vs. control): blue/green − 1.5 to <−0.5/ downregulated, light green/yellow/orange >−0.5 to < 0.5/ no or minor effect, red/dark red > 0.5 to 1.5/ upregulated, white- not detectable (nd). Figure S3. *TCSn::GFP* activity in developing barley grains after treatment with NAA and BAP. Two independent transgenic lines (E4 and E7) were examined. Grains were treated either with synthetic auxin (NAA, 50 µM) or cytokinin (BAP, 5 µM) for 16 h. Transverse sections of grains at different developmental timepoints (3-5DAF) with magnification of the ETC region at the maternal-filial boundary, bars = 100 μm. Figure S4. Heat map display of transcript levels of barley TCS genes in grains and vegetative tissues under stress treatment (*Fusarium*, drought, cold, heat, light/dark). Expression levels (FPKM-values) are displayed by color code. Blue- low, yellow- intermediate, red- high transcriptional levels and white boxes indicating not expressed (FPKM < 1). *Abbreviations*: 3ADON – *Fusarium* strain 3ADON; YI – Young inflorescence (see Table S4 for details). Figure S5. HP cluster genes in selected wild barley and landraces compared to cultivar ‘Morex’. (A) Phylogenetic tree of HP cluster elements of seven barley accessions: cultivar Morex, wild barley WBDC133, WBDC184 and B1K-17-07, and landraces 10TJ18, HOR21599 and HOR495. Protein sequences of AHP1 from *Arabidopsis* and HP1 and HP2 from rice are used for comparison. (B) Table shows reduced number of HP cluster genes in wild barley and landrace accessions. Presence of HP cluster elements in the accessions; multiple copies highlighted. Methods S1. Details of preparation of a cell type/tissue-specific cDNA library.

Additional file 2: Supplementary Table S1. Gene IDs, DNA and protein sequences of barley TCS elements. Supplementary Table S2. Ortholog screening of TCS elements in barley pangenomes. Supplementary Table S3. Gene IDs and protein sequences of TCS elements from *Arabidopsis*, rice and transfer cell specific maize elements (TCRR). Supplementary Table S4. Sample abbreviations and descriptions for RNA-seq databases and qPCR. Supplementary Table S5. FPKM-values of TCS elements in vegetative barley tissues. Supplementary Table S6. FPKM-values of TCS elements in developmental stages of barley spike meristems. Supplementary Table S7. FPKM-values of TCS elements in barley inflorescences. Supplementary Table S8. FPKM/TPM-values of TCS elements in barley grain tissues. Supplementary Table S9. Relative expression of selected barley TCS elements in grain tissues. Supplementary Table S10. ΔΔCt-values of barley TCS elements after treatment with hormones. Supplementary Table S11. FPKM-values of TCS elements under stress conditions. Supplementary Table S12. List of oligonucleotides for qPCR and PCR screening.

## Data Availability

All data generated and analyzed for this study are included in the manuscript and its supplementary information files.
