# Supplementary material for: Genome-wide characterization of two-component system elements in barley enables the identification of grain-specific phosphorelay genes
Source: BMC Plant Biol. 2025 Feb 17;25:209. doi: 10.1186/s12870-025-06161-1 (PMC11831784; doi:10.1186/s12870-025-06161-1)
Supplement: Supplementary file 1 — Additional file 1: Figure S1. Phosphorylation sites in authentic and pseudo HPs and RRs. (A) Histidyl phosphorylation site in HPs (red) and alternative amino acid in PHPs (blue). (B) Aspartate phosphorylation site in RRs (red) and alternative amino acids in PRRs (blue). Figure S2. Altered expression of barley TCS elements after treatment with hormones. Grains (5DAF) were incubated for 16 h either with abscisic acid (ABA, 50 µM), synthetic auxin 1-Naphthaleneacetic acid (NAA, 50 µM), cytokinin 6-Benzylaminopurine (BAP, 5 µM) or the ethylene precursor 1-Aminocyclopropane-1-carboxylic acid (ACC, 10 µM). Color code illustrates ΔΔCt values corresponding to log2-fold change (hormone vs. control): blue/green − 1.5 to <−0.5/ downregulated, light green/yellow/orange >−0.5 to < 0.5/ no or minor effect, red/dark red > 0.5 to 1.5/ upregulated, white- not detectable (nd). Figure S3. TCSn::GFP activity in developing barley grains after treatment with NAA and BAP. Two independent transgenic lines (E4 and E7) were examined. Grains were treated either with synthetic auxin (NAA, 50 µM) or cytokinin (BAP, 5 µM) for 16 h. Transverse sections of grains at different developmental timepoints (3-5DAF) with magnification of the ETC region at the maternal-filial boundary, bars = 100 μm. Figure S4. Heat map display of transcript levels of barley TCS genes in grains and vegetative tissues under stress treatment (Fusarium, drought, cold, heat, light/dark). Expression levels (FPKM-values) are displayed by color code. Blue- low, yellow- intermediate, red- high transcriptional levels and white boxes indicating not expressed (FPKM < 1). Abbreviations: 3ADON – Fusarium strain 3ADON; YI – Young inflorescence (see Table S4 for details). Figure S5. HP cluster genes in selected wild barley and landraces compared to cultivar ‘Morex’. (A) Phylogenetic tree of HP cluster elements of seven barley accessions: cultivar Morex, wild barley WBDC133, WBDC184 and B1K-17-07, and landraces 10TJ18, HOR21599 and HOR49 [file 12870_2025_6161_MOESM1_ESM.pdf]

Additional file 1

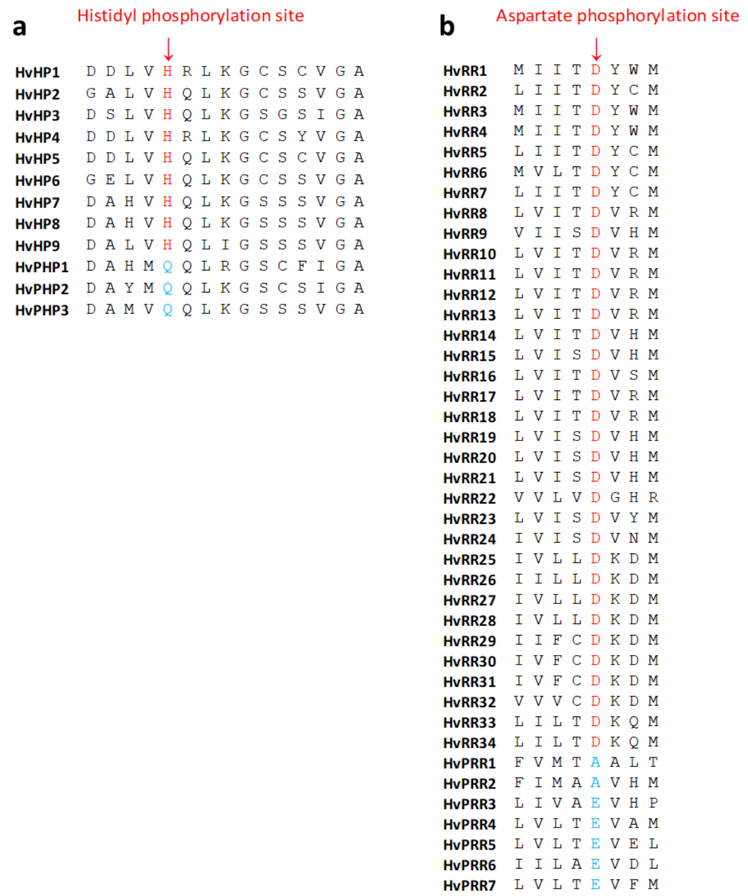

Figure S1. Phosphorylation sites in authentic and pseudo HPs and RRs. (a) Histidyl phosphorylation site in HPs (red) and alternative amino acid in PHPs (blue). (b) Aspartate phosphorylation site in RRs (red) and alternative amino acids in PRRs (blue).

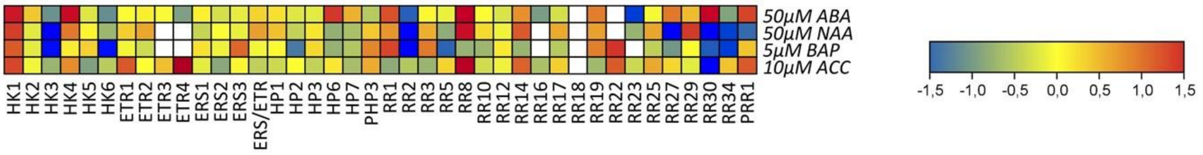

Figure S2. Altered expression of barley TCS elements after treatment with hormones. Grains (5DAF) were incubated for 16 h either with abscisic acid (ABA, 50 µM), synthetic auxin 1-Naphthaleneacetic acid (NAA, 50 µM), cytokinin 6-Benzylaminopurine (BAP, 5 µM) or the ethylene precursor 1-Aminocyclopropane-1-carboxylic acid (ACC, 10 µM). Color code illustrates ΔΔCt values corresponding to $\log_2$-fold change: blue/green -1.5 to <-0.5/ downregulated, light green/yellow/orange >-0.5 to <0.5/ no or minor effect, red/dark red >0.5 to 1.5/ upregulated, white not detectable (nd).

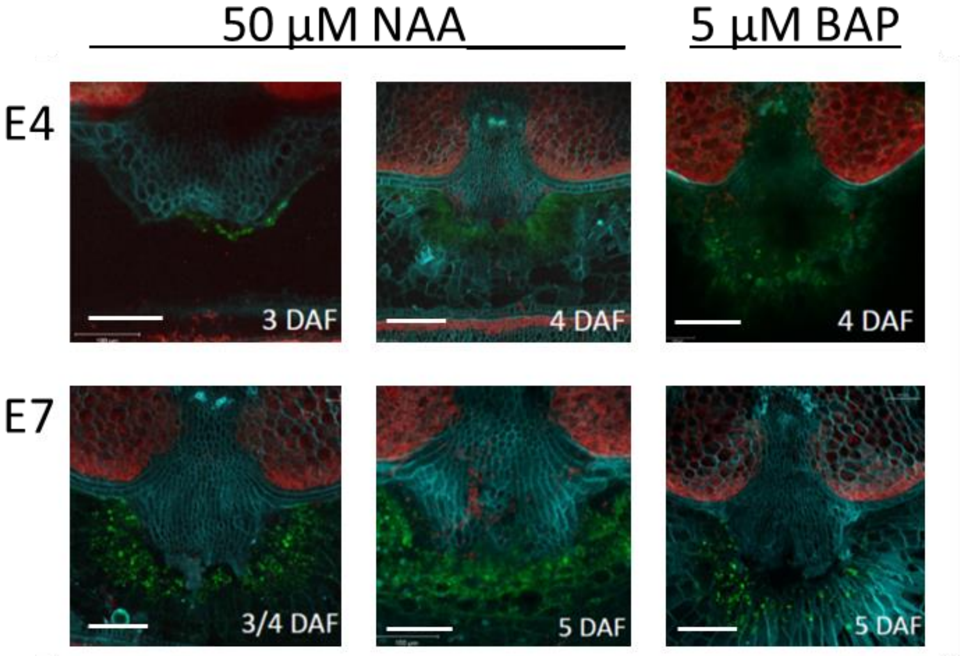

Figure S3. *TCSn::GFP* activity in developing barley grains after treatment with NAA and BAP. Two independent transgenic lines (E4 and E7) were examined. Grains were treated either with synthetic auxin (NAA, 50 µM) or cytokinin (BAP, 5 µM) for 16h. Transverse sections of grains at different developmental timepoints (3-5DAF) with magnification of the ETC region at the maternal-filial boundary, bars = 100 µm.

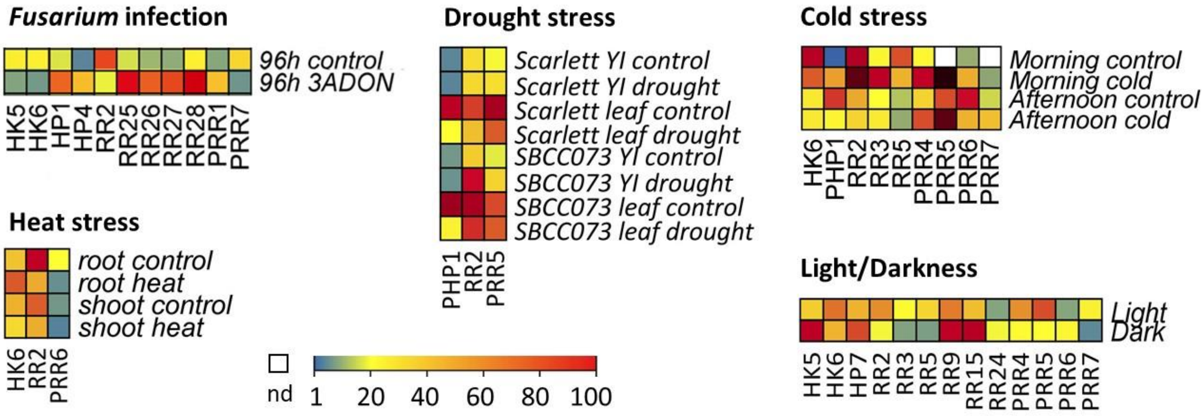

Figure S4. Heat map display of transcript levels of barley TCS genes in grains and vegetative tissues under stress treatment (*Fusarium*, drought, coldness, heat, light/dark). Expression levels (FPKM-values) are displayed by color code. Blue- low, yellow- intermediate, red- high transcriptional levels and white boxes indicating not expressed (FPKM< 1). *Abbreviations*: 3ADON – *Fusarium* strain 3ADON; YI – Young inflorescence (see Table S4 for details).

a

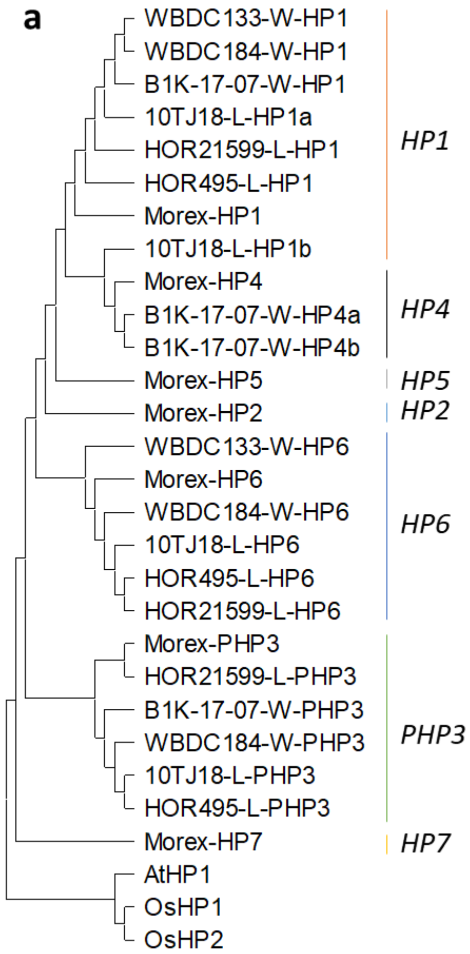

b

Accession	HP cluster genes
cultivar Morex	7
landrace 10TJ18	4 (2x HP1)
landrace HOR21599	3
landrace HOR495	3
wild barley WBDC133	2
wild barley WBDC184	3
wild barley B1K-17-07	4 (2x HP4)

Figure S5. HP cluster genes in selected wild barley and landraces compared to cultivar 'Morex'. (a) Phylogenetic tree of HP cluster elements of seven barley accessions: cultivar Morex, wild barley WBDC133, WBDC184 and B1K-17-07, and landraces 10TJ18, HOR21599 and HOR495. Protein sequences of AHP1 from Arabidopsis and HP1 and HP2 from rice are used for comparison. (b) Table shows reduced number of HP cluster genes in wild barley and landrace accessions. Presence of HP cluster elements in the accessions; multiple copies highlighted.

Methods S1. Details of preparation of a cell type/tissue-specific cDNA library

A tissue-specific cDNA library was generated in the 'Seed Development' group at the IPK Gatersleben as a resource for performing gene expression analysis of any gene of interest by qPCR. The library is composed of 33 LCM-isolated grain tissues with a focus on transport tissues within the grain (Fig. S6a, b), i.e. main vascular (MVB) and side vascular bundles (sVB); nucellar projection (NP) in maternal grain parts and the filial endosperm transfer cells (ETCs). The seed maternal tissues (SMATs) were further divided in the dorsal and ventral pericarp (PD, PV, boxed in Fig. S6b) and the green chlorenchyma (CHL); the filial endosperm tissues in the syncytial endosperm (SYNC, only 3 DAF), central endosperm (CE) and endosperm wings (WE). The developmental time frame covered the earliest stages of grain development until the grain filling period has been started (1 to 14 DAF, Fig. S6b). Sample collection

a

Designation	Sample description	Developmental Stage
sVB	side vascular bundle	1, 3, 5 DAF
MVB	main vascular bundle	1, 3, 5,7, 10, 14 DAF
NP	nucellar projection	3, 5,7, 10, 14 DAF
CHL	chlorenchyma	3, 5,7, 10, 14 DAF
ETC	endosperm transfer cells	3, 5,7, 10, 14 DAF
SYNC	syncytial endosperm	3 DAF
CE	central endosperm	5,7, 10, 14 DAF
WE	endosperm wings	5,7, 10, 14 DAF
PV	pericarp ventral	3, 6/7, 10, 12 DAF
PD	pericarp dorsal	3, 6/7, 10, 12 DAF
Stigma	stigma region of the grain	1, 3 DAF
Embryo R	embryo region of the grain	1, 3, 5,7, 10, 14 DAF
C	comple grain-Control (mock)	5 DAF
50µM ABA	comple grain+50µM ABA	5 DAF
50µM NAA	comple grain+50µM NAA	5 DAF
5µM BAP	comple grain+5µM BAP/CYT	5 DAF
10µM ACC	comple grain+10µM ACC/Eth	5 DAF

b

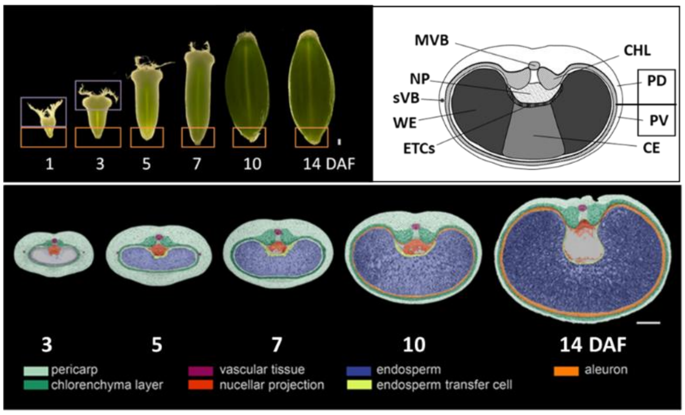

c

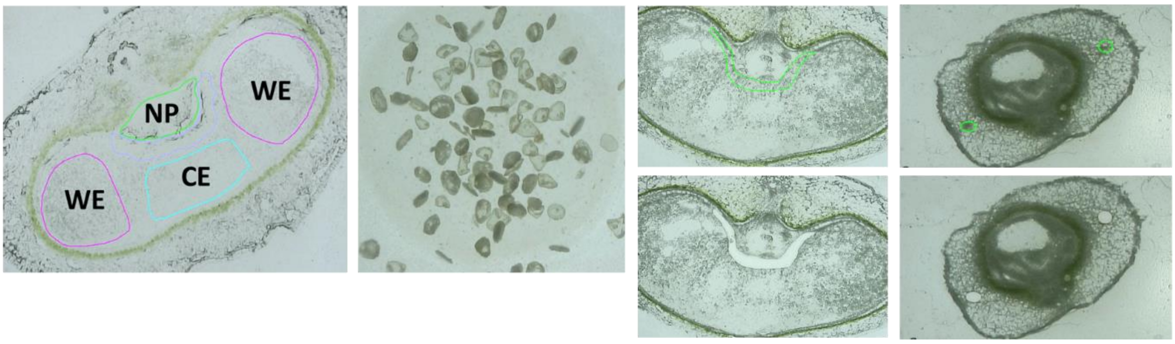

Fig. S6. Samples of a highly-resolved cDNA library for qPCR analysis. (a) Table lists sample descriptions; LCM-isolated samples are shaded in grey, manually dissected samples in blue and complete treated grains in green. (b) Representation of developmental stages and tissue types sampled for the cDNA library. Transverse sections with labelled grain tissues, schematized section from a grain at 7 DAF shows collected cell/tissue types. (c) Representative cryosections with marked tissues for LCM-based isolation. NP, CE and WE from a 7 DAF grain are marked by the software (left), targeting and sectioning of ETCs at 7 DAFs and VBs at 1 DAF (right panel), LCM sections are captured in a lid of an adhesive cap (middle).

was complemented by manually dissected regions of the grain: top region mainly composed of the stigma and bottom region containing the embryo. Further, complete grains (5 DAF) incubated with different hormones (Fig. S6a) are included.

Barley grains at different developmental stages were harvested, immediately frozen in liquid nitrogen and transferred to a cryostat (CryostarTM NX70, Thermo Fisher Scientific, Walldorf, Germany) cooled

down to −20°C. Using a razor blade, the middle part of the grains was cut out and glued onto the sample plate by using Tissue-Tek® O.C.T™ compound (Sakura Finetek Europe B.V., Zoeterwoude, Netherlands). Serial transverse sections of 20 µm thickness were cut and mounted on PEN membrane slides (PALM, Carl Zeiss Micro Imaging GmbH, Jena, Germany). PEN membrane slides were stored for 7 days in the cryostat at −20°C until complete dryness. Prior to microdissection, dry cryosections were adapted to room temperature.

The LCM procedure using the PALM® MicroBeam laser system (PALM) is exemplarily shown for sVBs at 1 DAF and ETCs from grains at 7 DAF (Fig. S6c). Firstly, the tissue of interest is marked by using the PALM® RoboSoftware, after targeting the tissue is cut by the laser and catapulted by a high-pressure laser beam in the lid of an adhesive cap (Adhesive Cap 500 opaque, Zeiss, Jena, Germany). Between 30 and 300 dissected cell types/tissues were captured for one sample type.

RNA was extracted using the Absolutely RNA Nanoprep Kit (Agilent, Waldbronn, Germany) and total RNA was amplified by one round of T7-based mRNA amplification using the MessageAmp aRNA Kit (Invitrogen, Carlsbad, CA, USA) to generate antisense RNA (aRNA). After measurement of concentrations by NanoDrop™ 2000 (Thermo Fisher Scientific, Walldorf, Germany) and quality assessment of aRNA populations by using an Agilent 2100 Bioanalyzer (Agilent, Waldbronn, Germany), 300 ng of aRNA was used for first strand cDNA synthesis of each sample. SuperScript® III First-Strand Synthesis System for RT-PCR (Invitrogen, Carlsbad, USA) with random priming was used according to the manufactureŕs instructions to generate normalized cDNA samples.
